# Rapid reversal of innate immune dysregulation in blood of patients and livers of humanized mice with HCV following DAA therapy

**DOI:** 10.1371/journal.pone.0186213

**Published:** 2017-10-17

**Authors:** Matthew A. Burchill, Justin A. Roby, Nanette Crochet, Megan Wind-Rotolo, Amy E. Stone, Michael G. Edwards, Rachael J. Dran, Michael S. Kriss, Michael Gale, Hugo R. Rosen

**Affiliations:** 1 Division of Gastroenterology and Hepatology, Hepatitis C Center, Department of Medicine, University of Colorado Denver (UCD), Aurora, Colorado, United States of America; 2 Center for Innate Immunity and Immune Disease, University of Washington, Seattle, Washington, United States of America; 3 Department of Immunology, University of Washington, Seattle, Washington, United States of America; 4 Bristol-Myers Squibb, Exploratory Clinical and Translational Research, Lawrenceville, New Jersey, United States of America; 5 Bioinfo Solutions LLC, Parker, Colorado, United States of America; University of Sydney, AUSTRALIA

## Abstract

Chronic hepatitis C virus (HCV) infection results in sustained immune activation in both the periphery and hepatic tissue. HCV infection induces innate immune signaling that is responsible for recognition of dsRNA, leading to activation of transcription factors and production of Type I and III IFNs, as well as pro-inflammatory cytokines and chemokines. Continued activation of host-immune mediated inflammation is thought to contribute to pathologic changes that result in progressive hepatic fibrosis. The current standard treatment for chronic HCV infection is directly-acting antivirals (DAAs), which have provided the unique opportunity to determine whether successful, rapid treatment-induced eradication of viral RNA normalizes the dysregulated antiviral innate immune response in patients chronically infected with HCV.

**Results:**

First, in patients receiving two different combinations of DAAs, we found that DAAs induced not only rapid viral clearance, but also a re-setting of antiviral immune responses in the peripheral blood. Specifically, we see a rapid decline in the expression of genes associated with chronic IFN stimulation (IFIT3, USP18, IFIT1) as well as a rapid decline in genes associated with inflammation (IL1β, CXCL10, CXCL11) in the peripheral blood that precedes the complete removal of virus from the blood. Interestingly, this rapid reversal of innate immune activation was not seen in patients who successfully clear chronic HCV infection using IFN-based therapy. Next, using a novel humanized mouse model *(Fah*^*-/-*^*RAG2*^*-/-*^*IL2rg*^*null*^—FRG), we assessed the changes that occur in the hepatic tissue following DAA treatment. DAA-mediated rapid HCV clearance resulted in blunting of the expression of proinflammatory responses while functionally restoring the RIG-I/MAVS axis in the liver of humanized mice.

**Conclusions:**

Collectively, our data demonstrate that the rapid viral clearance following treatment with DAAs results in the rebalancing of innate antiviral response in both the peripheral blood and the liver as well as enhanced antiviral signaling within previously infected hepatocytes.

## Materials and methods

### Patient demographics

*DAA cohort 1*. Patients were selected from a randomized, phase 2a trial as previously described [1, 2]. *DAA cohort 2*. Patients were enrolled in a prospective study at the University of Colorado-Anschutz Medical Campus (Aurora, CO) and the Veterans Affairs Hospital (Denver, CO). *IFNα/Ribavirin cohort*. Patients were selected from our repository as previously described [3, 4]. All patients provided written informed consent and the study was approved by appropriate institutional review boards at the University of Colorado—Anschutz and Bristol-Myers Squibb.

#### PBMC and Paxgene collection

*DAA cohort 1*. One preparation tube (CPT) and two Paxgene tubes were collected at each time point and processed. Cells were washed with RPMI and frozen with FBS/10% DMSO. Two aliquots of PBMCs were obtained for each CPT tube. *DAA cohort 2*. Sixteen preparation tubes (CPT) were collected prior to the start of treatment and at the post-treatment time point. Eight CPT tubes were collected at time points indicated during the course of treatment. CPT tubes were processed and PBMCs were isolated according to manufacturer protocols. Cells were frozen with FBS/10% DMSO at a concentration of 1x10^7^/ML.

#### RNA analysis

PBMCs were thawed in RPMI containing 10% human serum AB, washed, enumerated and mRNA was isolated using the RNeasy Mini kit (Qiagen, Germantown, MD). mRNA was isolated from Paxgene tubes using the Paxgene Blood RNA kit (Qiagen). For microarray analysis 100ng of mRNA was labeled using the WT Plus Reagent Kit (thermofisher). The labeled cDNA was hybridized to Human Gene 2.0 Arrays (Affymetrix, Santa Clara, CA) for 16 hours at 45°C in a Genechip Hybridization Oven 640. Chips were washed and stained in a Genechip Fluidics Station 450 and were scanned in a Genechip Scanner 7G as previously described[5]. Partek Genomics Suite (PGS) v6.6 (Partek, St Louis, MO) was used to quantitate gene expression to log2 values using RMA background correction with quantile normalization and medial polish for probeset summarization. A paired t-test was used to generate p-values for all genes (53,617 transcripts) comparing pre- vs post- DAA treatment. The normalized expression values used in the statistical and bioinformatics analysis, as well as the original raw visual data files used to calculate these values, have been deposited in the publicly accessible database Gene Expression Omnibus (http://www.ncbi.nlm.nih.gov/geo/) under the accession number GSE104597. For the interferon dataset (GEO accession # GSE11342), all raw image files for Human Genome U133A Affymetrix arrays were downloaded from NCBI and gene expression quantitated and normalized using the same method as the current study. A paired t-test was used to calculate p-values for all genes (22,283 transcripts) comparing pre- vs. post- interferon treatment. For RT-PCR, 1ug of mRNA was converted to cDNA using the QuantiTect Reverse Transcription kit (Qiagen) according to manufacturer protocol. cDNA was then analyzed for gene expression levels using the indicated primers (Qiagen)

#### Analysis of NFkB response

Patient PBMCs harvested prior to, and subsequent to DAA treatment were lysed with radioimmunoprecipitation assay buffer (50 mM Tris pH 7.4, 150 mM NaCl, 1% (v/v) Triton X-100, 0.5% (w/v) Na-deoxycholate, 250 nM okadaic acid, 1:100 Phosphatase Inhibitor Cocktail Set II (Calbiochem, USA), 1:100 Protease Inhibitor Cocktail P8340 (Sigma, USA)) and 14 μg total protein was resolved on a 4–20% Criterion^™^ TGX^™^ polyacrylamide gel (BioRad, USA). Following electrophoresis, proteins were transferred onto nitrocellulose membranes and blocked overnight at 4°C in Odyssey Blocking Buffer (TBS) (LI-COR, USA). Antibodies used for analysis were mouse anti-NF-κB p65 (L8F6) (Cell Signaling Technology, USA), rabbit anti-NF-kB p65 (phospho S536) (EP2294Y) (Abcam, USA), and mouse anti-Actin (clone C4) (Millipore, USA), with secondary antibodies peroxidase-conjugated goat anti-mouse IgG, donkey anti-rabbit IgG, and Alexa Fluor 790-conjugated donkey anti-mouse IgG (Jackson ImmunoResearch, USA). Pixel intensity of imaged immunoblot bands were quantified using NIH ImageJ software, and activation of the NF-kB pathway was inferred by dividing the value of phospho-NF-kB p65 by the value of total NF-kB p65 for each patient sample. To reduce variance between repeat experiments on different membranes, the ratio derived from samples in the first lane of each gel were arbitrarily set to one and all remaining samples from that experiment were scaled to match. Statistical analysis was performed using a two-tailed Wilcoxon matched pairs test on Prism 5 software (Graph Pad).

#### Mice

FRG mice were obtained from Yecuris (Tualatin, OR) and maintenance of human liver chimerism was performed as described [6, 7]. Degree of human hepatocyte engraftment was measured using a human albumin ELISA (Bethyl Laboratories, Montgomery, Tx). All mice utilized in experiments had >90% engraftment of human hepatocytes. For infection, mice were intravenously administered serum from a patient chronically infected with HCV geneotype 1a. The infectious dose of HCV was 1.53x10^6^ IU. For DAA treatment mice were given Asunaprevir (0.3 mg daily), Declatasvir (0.5mg BID), and Sofosbuvir (2mg daily) or DMSO as a control via oral gavage. During treatment infectious load was measured in the peripheral blood every 24 hours via craniofacial bleed. Mice were monitored daily for signs of distress and no mice had to be euthanized prior to completion of the study. Following a 14-day course of treatment mice were euthanized with CO_2_ followed by cervical dislocation and livers were fixed in formalin for 30min at 25°C and embedded in paraffin. This study was carried out in accordance with the recommendations in the Guide for the Care and Use of Laboratory Animals of the National Institutes of Health. All experiments were approved by the institutional animal care and use committee (IACUC) at the University of Colorado Anschutz medical campus.

### Immunohistochemistry and confocal microscopy

For sections of paraffin-embedded liver biopsies, we used the following procedure involving 4 major steps, including deparaffinization of tissue, antigen retrieval, tissue permeabilization, and immunostaining. Slide-mounted paraffin-embedded biopsy tissue was deparaffinized by first placing slides on a 58°C heat block for 5 minutes in order to preheat them to the working temperature. Preheated slides were then placed in a glass Coplin jar containing 1x EZ Dewax solution (Biogenex) and were incubated for 5 minutes at room temperature. Slides were then placed in xylene and incubated for 5 minutes at room temperature followed by sequential 5 minute incubations in a Coplin jar each containing 100% ethanol, 95% ethanol, 70% ethanol, and distilled ultrapure water. Slides were placed in IFA buffer (PBS containing 0.05% TWEEN-20) for 5 minutes, and were then subjected to antigen retrieval. For antigen retrieval of deparaffinized slide-mounted tissue, slides were placed in a plastic Coplin jar containing 1x AR10 solution (Biogenex), and a vented lid was placed on the jar. The jar was then set inside a microwave oven and incubated with an oven setting on high while constantly monitoring through the oven door to define the first point of boiling of liquid within the jar. The oven power was turned off when the first point of boiling was observed, the liquid removed from the jar and the jar re-filled with 1x AR10 solution maintained at 4°C. The jar (containing the slides) was then allowed to cool for 10 minutes at room temperature. This cycle of heating and cooling was repeated, after which the jar was allowed to cool at room temperature for 30 minutes. Slides were then removed from the jar and rinsed sequentially with distilled ultrapure water and 1X PBS. Tissues were permeabilized by placing slides in a Coplin jar containing PBS/1.0% TritonX-100 for 5 minutes at 25°C. Slides were placed in 1X PBS for 5 minutes then air dried. For immunostaining, slides were first incubated in PBS containing 10% normal goat serum for 1 hour. Primary antibody was applied and the slides were incubated for one hour at 25°C. The slides were rinsed three times 5 minutes each with IFA buffer followed by application of secondary antibody coupled to Alexa488 or Alexa 594 fluorophores (Molecular Probes). After a 1 hour incubation at 25°C, the slides were rinsed three times with IFA buffer (PBS containing 0.05% TWEEN-20), allowed to dry, and cover slips were mounted using Prolong Gold mounting medium (ThermoFisher Scientific) for 24 hours before imaging. Tissues were imaged using a Nikon Eclipse TE2000 inverted microscope with the Nikon C1 laser scanning confocal module utilizing a 10 mW Argon laser emitting light at 488 nm wavelength, a 1 mW HeNe laser emitting light at 543 nm wavelength, and a 5 mW HeNe laser emitting light at 633 nm wavelength. Digital images were collected as 0.2 μM optical sections and were processed using Nikon EZ-C1 Software v.3.40. Multiple images were collected for each sample analyzed. The primary antibodies used were:

Hepatitis C virus (NS3) (1:100, Novocastra Laboratories Ltd. NCL-HCV-NS3), IPS-1 (AT107) (MAVS) (1:100, Alexis 210-929-0100), IFITM1 (1:100, ProteinTech Group Inc. 60074-1-1g)

IRF3 (md) (1:100, kindly provided by *Michael David*). The secondary antibodies used were:

Alexa 594 goat anti-rabbit IgG (1:100, ThermoFisher A-11012), Alexa 488 goat anti-mouse IgG (1:100, ThermoFisher A-11001), DAPI (1:1000, ThermoFisher D1306)

Statistical analysis and graphical illustration of data unless otherwise mentioned was performed using Prisim 5 (GraphPad, San Diego, CA).

## Introduction

Chronic infection with hepatitis C Virus (HCV) results in chronic upregulation of many genes associated with innate immune activation [8]. This chronic stimulation of the innate immune response and subsequent activation of hepatic stellate cells are the primary driver of liver inflammation and ultimately cirrhosis [9]. HCV stimulates the innate immune response through multiple mechanisms including activation of TLRs 2,3,4,7/8, and 9 [10]. Therefore, HCV infection can induce activation of virtually every human innate immune cell in both the liver and the peripheral blood, which results in the increased expression of many genes and proteins associated with inflammation and consequent liver fibrosis. For example, chronic HCV infection results in the accumulation of active IL1β in the serum of patients [11]. Additionally, IL1β is associated with the activation of HSCs [12, 13], ultimately leading to liver fibrosis. This chronic inflammation caused by IL1β as well as other factors has established that chronic activation of the innate immune response by HCV is critical for the progression to end-stage liver disease. Previous standard of care therapy with IFNα/ribavirin utilized the innate immune response to rid the body of HCV infection in part by boosting the innate immune compartment. However, this treatment regimen had limited success [14], immunomodulatory effects on the adaptive immune system which are critical for viral clearance, and well-documented and significant side effects [15–17]. The recent advances in therapy for HCV, via DAAs, has allowed for the dissection of how rapid HCV clearance alters innate immune activation and ultimately liver disease progression in the absence of the immunomodulatory effects of IFN.

In this report, we document how rapid clearance of HCV resets innate immune activation via DAA therapy, how treatment with DAA therapy differs from IFNα/ribavirin therapy in its effect on innate immune activation, and finally how DAA therapy alters innate immune activation in the hepatic compartment of humanized mice. Our observations define the molecular changes in the peripheral blood that are associated with successful clearance of HCV with novel IFN-free DAA therapies.

## Results

### DAA treatment results in rapid downregulation of the innate immune signature in the peripheral blood of chronically infected patients

**Fig 1:**
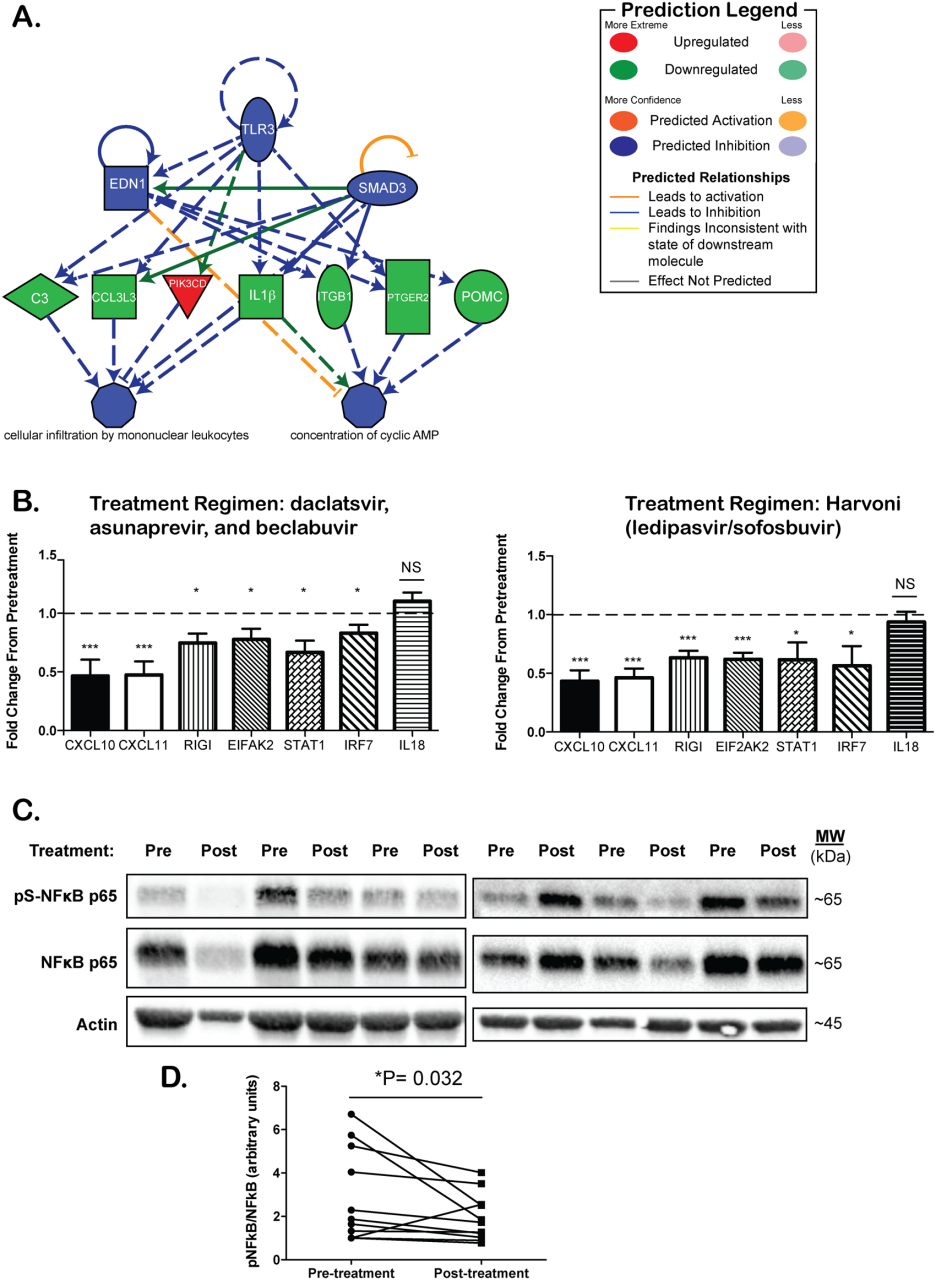
DAA therapy results in a reduction in innate immune activation in the peripheral blood. **(A)** Graphical representation of the measured or predicted activation state (IPA) of innate immune signaling molecules at the end of DAA therapy compared to pretreatment. Green is inhibited whereas red represents increased expression. **(B)** Semi-quantitative RT-PCR of select genes associated with HCV-induced inflammation from PBMCs treated with two different regimens of DAA at end of treatment compared to pretreatment in DAA cohort 1 (n = 18, top) or DAA cohort 2 (n = 11, bottom). **(C)** Western blot analysis of the levels of pS-NFκB, and NFκB. Top: representative images of 6 patient samples prior to (Pre) and twelve weeks following completion of DAA therapy (Post) in DAA cohort 2. **(D)** Quantification of the ratio of pS-NFκB to NFκB (right) prior to treatment (circles) and at twelve weeks following the end of treatment (squares). Lines represent individual patient samples. *P = 0.03, Wilcoxon matched-pairs signed rank test. N = 11 patients.

We performed microarray mRNA expression profiling in PBMCs of patients who received a three-drug regimen of DAAs: daclatasvir (NS5A inhibitor), asunaprevir (NS3 protease inhibitor), and beclabuvir (NS5B inhibitor). The median age was 55 years old; 88.9% of patients were men. The median pre-treatment viral level was 488,859 IU/ML and the median fibrotest score was 0.47; all patients experienced an SVR12 (S1A Table). DAA-mediated cure of chronic HCV infection resulted in significant transcriptional changes; specifically, when analyzing the top genes that are either increased or decreased in the peripheral blood at the end of DAA therapy, we observe a re-setting of innate immune antiviral responses and an increase in adaptive immune responses in the peripheral blood (Table 1). These transcriptional changes were confirmed using RT-PCR (S1 Fig). Using IPA-ingenuity software (Qiagen), we found that in addition to direct transcriptional changes, DAA therapy results in the predicted reduction in activation state of several upstream regulators of innate immune signaling such as Toll-like receptors (TLRs), chemokines, IFN-α2, and interleukin-1 (Table 2). Thus, after examination of innate immune signaling we observed significant dampening of innate immune responses both in gene expression levels (e.g., IL1β) and predicted activation state (e.g., TLR3) (Table 2 and Fig 1A).

**Table 1:** Top transcriptional changes in the peripheral blood of patients receiving DAA therapy. Fold changes and p values are a paired comparison between end of treatment and pretreatment.

Symbol	Entrez Gene Name	Affymetrix/Entrez Gene	Exp p-value	Exp Fold Change
TRAC	T-cell receptor alpha constant	16781877	8.69E-03	1.978
TRAT1	T cell receptor associated transmembrane adaptor 1	16943719	5.48E-03	1.94
CD28	CD28 molecule	16889807	9.03E-03	1.91
GCNT4	glucosaminyl (N-acetyl) transferase 4, core 2	16997275	1.37E-03	1.83
YME1L1	YME1 like 1 ATPase	16782028	3.28E-03	1.809
RCAN3	RCAN family member 3	16660810	9.89E-03	1.749
TPRG1L	tumor protein p63 regulated 1-like	16658184	6.87E-03	1.661
LRIG1	leucine-rich repeats and immunoglobulin-like domains 1	16955939	8.76E-03	1.558
ITM2A	integral membrane protein 2A	17112364	6.76E-03	1.555
NBR2	neighbor of BRCA1 gene 2 (non-protein coding)	16834566	1.62E-03	1.545
ANXA5	annexin A5	16979482	1.73E-03	-1.644
CD300C	CD300c molecule	16848593	7.15E-03	-1.645
SIGLEC14	sialic acid binding Ig-like lectin 14	16874922	7.44E-03	-1.687
MAFB	v-maf avian musculoaponeurotic fibrosarcoma oncogene homolog B	16919242	7.32E-03	-1.693
NFIL3	nuclear factor, interleukin 3 regulated	17095703	5.99E-03	-1.82
FFAR2	free fatty acid receptor 2	16861030	3.77E-03	-1.934
EREG	epiregulin	16967843	3.92E-03	-1.996
SPRY2	sprouty RTK signaling antagonist 2	16780069	1.23E-03	-2.01
IL1B	interleukin 1 beta	16901986	5.94E-03	-2.144
IFIT3	interferon induced protein with tetratricopeptide repeats 3	16707184	8.82E-03	-2.846

**Table 2:** Predicted changes in activation status of upstream regulators (IPA) in the peripheral blood of patients receiving DAA therapy.

Upstream Regulator	Upstream Regulator	Predicted Activation State	Activation z-score	p-value
phorbol myristate acetate	chemical drug	Inhibited	**-2.486**	2.75E-04
TNFSF11	cytokine	Inhibited	**-2.557**	4.37E-04
Ccl2	cytokine	Inhibited	**-2.19**	8.98E-04
NFKBIA	transcription regulator	Inhibited	**-2.264**	2.33E-03
LIF	cytokine	Inhibited	**-2.172**	2.63E-03
JAK1	kinase	Inhibited	**-2**	2.97E-03
E. coli B4 lipopolysaccharide	chemical toxicant	Inhibited	**-3.22**	3.08E-03
TLR3	transmembrane receptor	Inhibited	**-2.922**	3.73E-03
Salmonella enterica serotype abortus equi lipopolysaccharide	chemical toxicant	Inhibited	**-2.178**	4.24E-03
IFNA2	cytokine	Inhibited	**-2.277**	6.18E-03
SMARCA4	transcription regulator	Inhibited	**-2.538**	6.74E-03
lipopolysaccharide	chemical drug	Inhibited	**-3.167**	9.08E-03
5-O-mycolyl-beta-araf-(1->2)-5-O-mycolyl-alpha-araf-(1->1')-glycerol	chemical—endogenous non-mammalian	Inhibited	**-2.236**	9.09E-03
EDN1	cytokine	Inhibited	**-2.571**	9.17E-03
IL1B	cytokine	Inhibited	**-3.588**	9.37E-03
NUPR1	transcription regulator	Inhibited	**-2.84**	9.57E-03
GNRH1	other	Inhibited	**-2**	1.05E-02
S100A9	other	Inhibited	**-2.573**	1.06E-02
TLR9	transmembrane receptor	Inhibited	**-2.778**	1.50E-02
IL1	group	Inhibited	**-2.459**	2.95E-02
MYCN	transcription regulator	Activated	2.195	0.164
sirolimus	chemical drug	Activated	2.2	1
U0126	chemical—kinase inhibitor	Activated	2.395	0.297
PD98059	chemical—kinase inhibitor	Activated	3.075	0.0361

Fold changes and p values are a paired comparison between end of treatment and pretreatment.

To confirm these transcriptional changes and determine whether these were unique to the DAA regimen used or a common transcriptional signature associated with DAA-mediated rapid HCV clearance, we performed RT-PCR on PBMC from the cohort above (S1A Table) and a cohort treated with ledipasvir-sofosbuvir (S1B Table). We found remarkably similar transcriptional changes regardless of treatment regimen. In both IFN-free treatment regimens, we found statistically significant decreases in C-X-C motif chemokine (CXCL)-10 and CXCL11 and reduced expression of the innate immune signaling molecules RIG-I, EIF2AK2, STAT1 and IRF7 (Fig 1B). Therefore, the rapid dampening of innate immune activation following rapid viral clearance with IFN-free DAA therapy is independent of the treatment regimen utilized. In addition to dampening of the transcriptional activation of innate immune genes in the peripheral blood of patients treated with DAA therapy, we observed a statistically significant reduction in the phosphorylation of the NFκB p65 subunit protein in the peripheral blood following DAA therapy (Fig 1C and 1D). This result is consistent with the predicted downregulation of NFκBIA activity revealed in Table 2. Thus, rapid viral clearance with DAA therapy results in a rapid reduction in innate immune activation at both the transcriptional and post-translational level in the peripheral blood.

**Fig 2:**
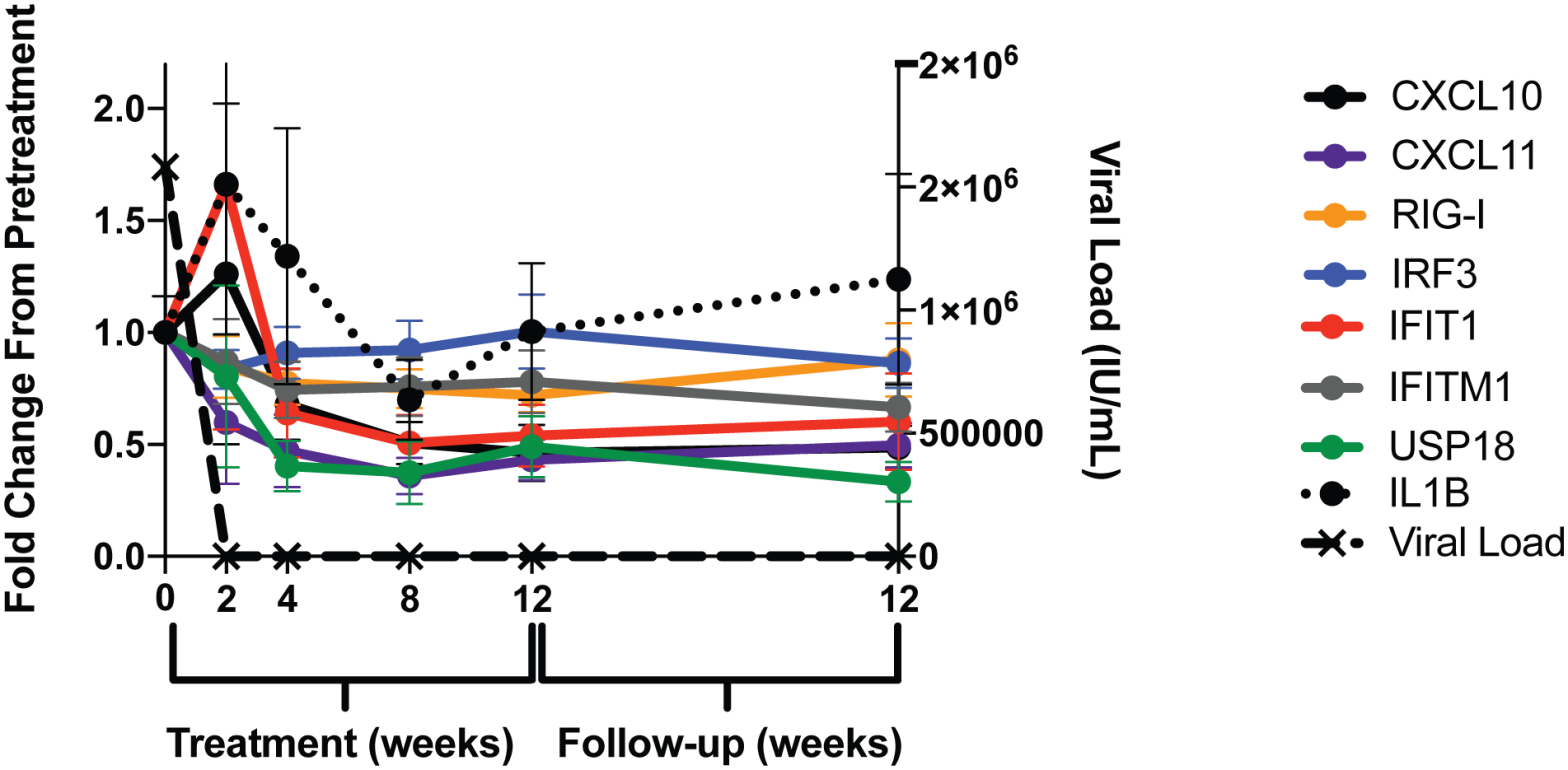
DAA therapy induces rapid suppression of some but not all antiviral signaling molecules. Kinetic analysis of transcriptional levels of CXCL10 (black), CXCL11 (purple), RIG-I (yellow), IRF3 (blue), IFIT1(ISG56) (red), IFITM1 (grey), USP18 (green), IL1β (black circles, dotted line) and plasma viral load (black crosses with black dashed line) from PBMCs in DAA patient cohort 2 (n = 11).

Next, we examined the kinetics of these transcriptional changes relative to viral decline in patients receiving DAAs. Using this longitudinal analysis we found that both CXCL10 and CXCL11 mirrored the rapid decline of HCV in the peripheral blood. Moreover, we found the expression of genes directly associated with innate immune sensing of IFN, such as ISG56 (IFIT1) and USP18, were rapidly decreased (Fig 2). In contrast, changes in RIG-I lagged behind and IRF3 expression did not decrease, suggesting distinct patterns of innate immune gene expression following DAA therapy. Interestingly, the expression of IL1β over the course of DAA therapy in patient cohort 2 was highly variable (perhaps related to different frequencies on monocytes), and thus at a global level, we did not detect a significant decrease in IL1β transcription in this cohort. These findings contrast with the significant decrease in IL1β transcription in the peripheral blood of patient cohort 1 by both microarray (Tables 1 and 2) and RT-PCR (S1 Fig). These data suggest that while both regimens of DAAs used in this study have the same clinical endpoint (cure of HCV), regimens may have slightly differing effects on the transcriptional signature in the peripheral blood. Taken together, these findings confirm and expand other published data [18], demonstrating IFN-free therapy can rapidly reverse the activation of some, but not all aspects of the innate immune response in the peripheral blood.

### Differential mRNA expression between DAA and IFN/ribavirin therapy

**Fig 3:**
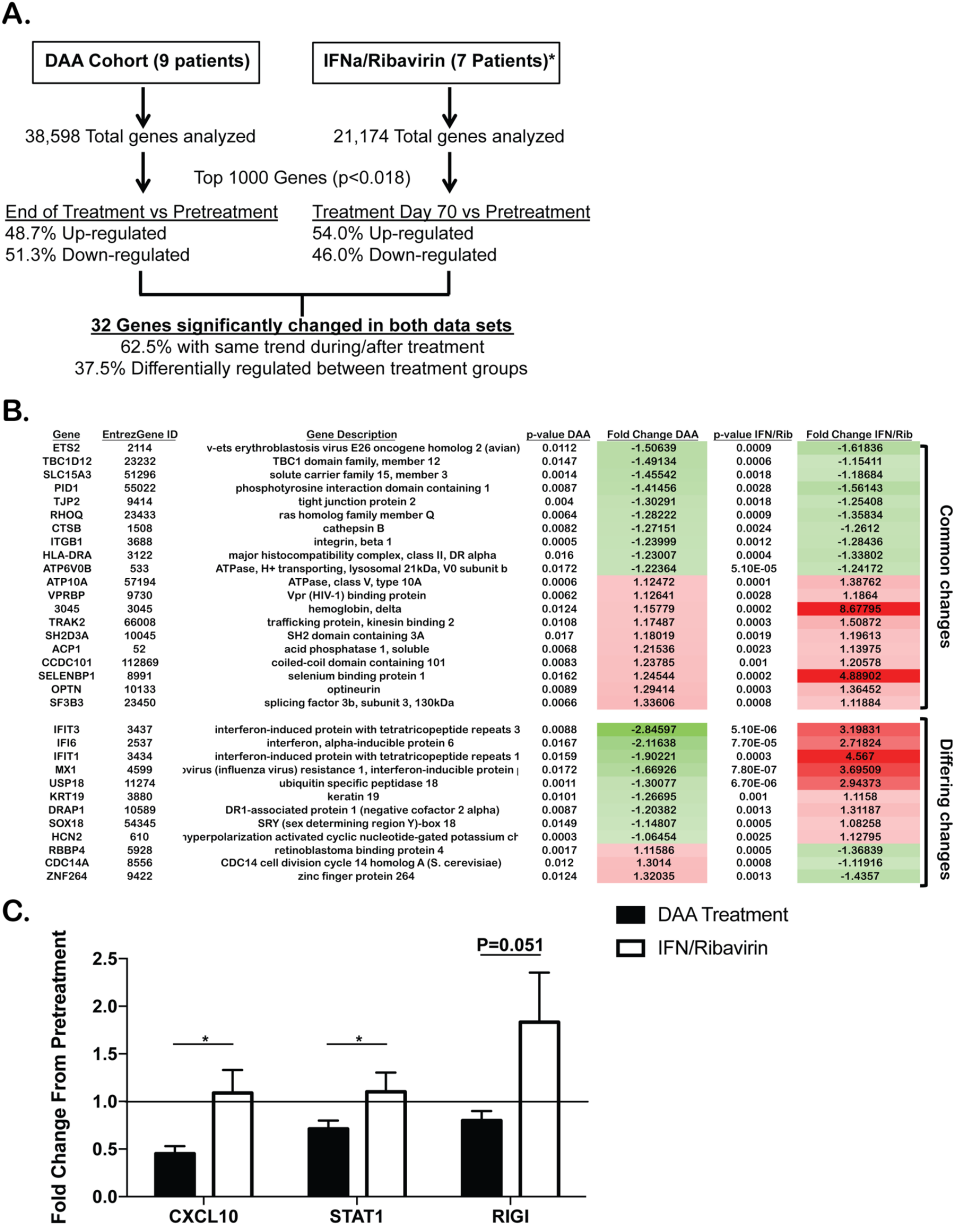
Differential transcriptional changes are associated with IFN-free DAA therapy and IFN/Ribavirin therapy. **(A)** Representation of the shared and different transcriptional changes in DAA therapy (EOT) compared to IFNα/Ribavirin therapy (wk 10) (19). The top 1000 genes statistically significantly changed from pretreatment in both data sets were compared to the expression of the given gene in the other treatment cohort. **(B)** List of the 32 genes that were statistically changed from post treatment to pretreatment in both data sets. **(C)** Quantitative RT-PCR comparing fold change from pretreatment at twelve weeks following DAA therapy (Black Bars) and twenty-four weeks following IFNα/Ribavirin therapy (White bars). P value represents comparison between both treatments. P<0.05, ***P<0.001, NS = Not significant. N = 8-11patients per group.

To determine which transcriptional changes are strictly associated with DAA therapy versus changes that merely are associated with viral cure, we utilized a published cohort of individuals treated with IFNα/ribavirin. DAA treatment directly interferes with the HCV replication cycle without altering the immune responses, in contrast to the use of IFN/ribavirin which has known immunomodulatory effects [10]. Therefore, we hypothesized that DAA therapy in the absence of IFN/ribavirin results in rapid reversal of HCV-induced chronic innate immune activation. To address the effects of these different regimens on innate immune activation, we directly compared our microarray data to the published microarray results of the Virahep-C cohort [19]. To control for potential differences in the rate of viral clearance between DAA therapy and IFN/ribavirin we focused our analysis on the seven patients with a rapid and sustained virologic responses. The Virahep-C time points for which microarray data was available was at pre-treatment and at 10 weeks of therapy (Fig 3A). When comparing the top 1000 genes that were significantly changed from pretreatment in both data sets, we found that only 32 (3.2%) genes were significantly different in both data sets with only 20 (62.5%) of those genes having a shared transcriptional change (Fig 3A). Comparison of these patients with end of DAA treatment patients (week 12) revealed many striking differences. Specifically, the dampening of the antiviral immune response seen with DAA therapy is not evident at 10 weeks of IFN/ribavirin therapy-even though these patients had cleared the virus (Fig 3B). As expected, a majority of the transcriptional changes between IFN-free DAA therapy and IFN/Ribavirin therapy are either directly or indirectly related to IFN treatment. To further document how the transcriptional changes following DAA therapy are represented in the peripheral blood of patients treated with IFNα/Ribavirin, we interrogated the 520 genes that were statistically different (p<0.01) from end of treatment to pretreatment in the DAA cohort. From this gene set we found 214 genes that were also measured in the IFNα/Ribavirin data set (S2 Fig). Using this methodology, we were able to validate the similarities and differences in gene expression between DAA and IFNα/Ribavirin therapy (S2A Fig). Additionally, when examining significant differences between these genes, we find that a vast majority of genes that are statistically different following DAA therapy are not statistically changed in the IFNα/Ribavirin data set (S2B Fig).

Next, we compared the innate immune gene expression in the peripheral blood of our cohort of IFN-free DAA treated individuals twelve weeks after completion of therapy to the signature of IFN/Ribavirin-treated individuals twenty four weeks after the end of treatment because the Virahep-C patients for whom microarray data were available were still receiving IFN/Ribavirin which affects gene expression. This latter analysis allowed for the interrogation of differences in transcriptional signature between DAA therapy and IFN-containing regimens in the absence of direct effects of IFN therapy on transcriptional signature as well as the durability of these differences. Surprisingly, in contrast to the sustained dampening of innate immune signaling seen with DAA therapy we did not see reduction in genes such as CXCL10, STAT1 and RIG-I long after the end of IFN/Ribavirin treatment and clearance of the virus (Fig 3C). Taken together, these data suggest that treatment with DAAs results in the rapid and prolonged dampening of antiviral innate signaling while treatment with IFN/Ribavirin can still be associated with persistent activation of innate immune responses even long after clearance of virus.

### IFN-free DAA therapy results in the rapid reduction of innate immune transcriptional signature in the livers of humanized mice

**Fig 4:**
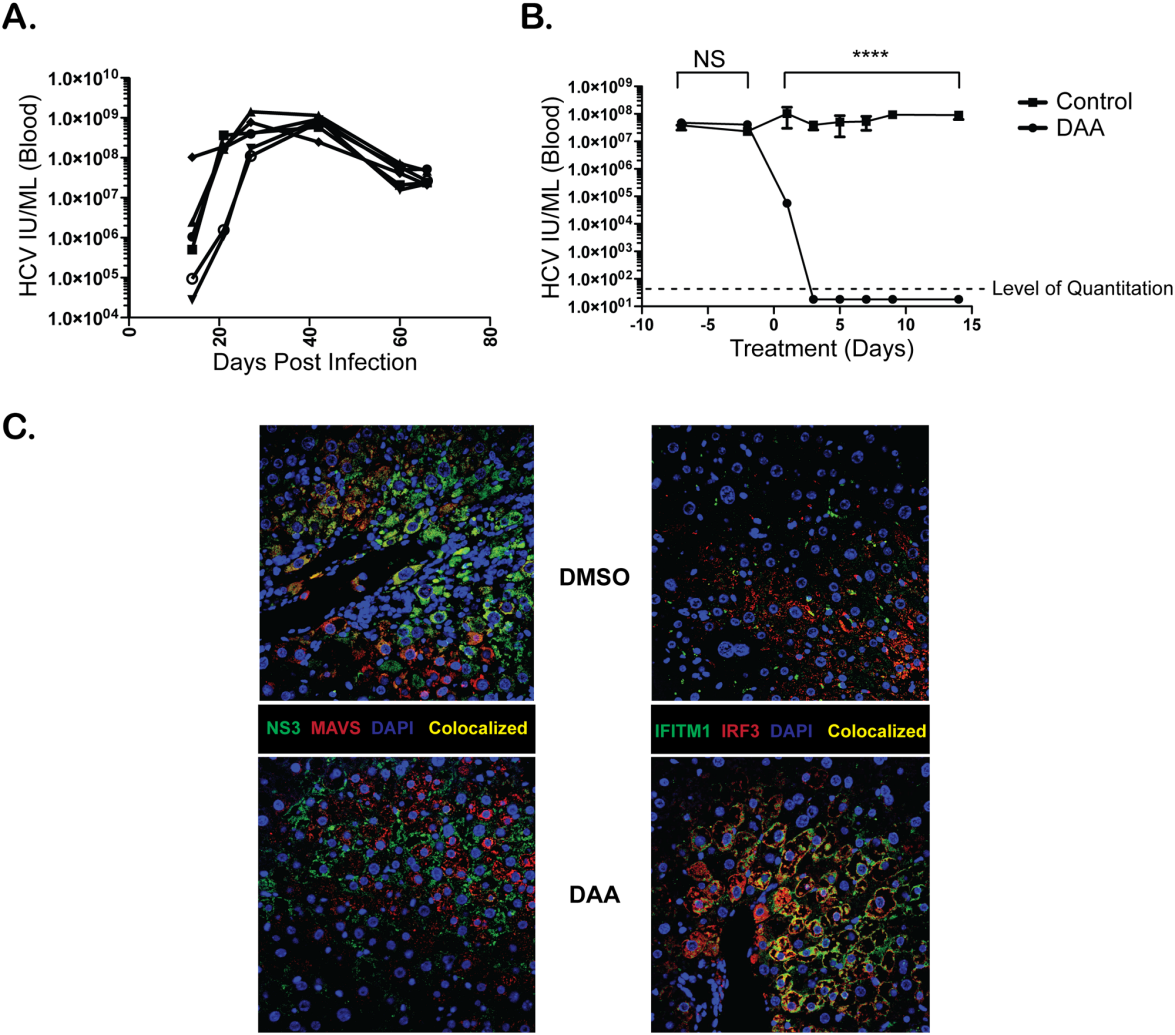
Restoration of HCV-suppressed antiviral signaling in the liver of humanized mice treated with DAAs. **(A)** Serum levels of HCV in mice injected with 1.4 x 10^6^ IU HCV intravenously. **(B)** HCV viral loads after treatment with Sofosbuvir (2.0mg daily), Daclatasvir (0.3mg daily), Asunaprevir (0.5mg twice daily) or DMSO vehicle control via oral gavage. **(C)** Protein expression of NS3, MAVS, IFITM1, and IRF3 following 14 days of DMSO treatment or DAA treatment in whole liver tissue via immunofluorescence staining. N = 3 mice per group. ****P<0.0001, NS = Not significant.

In order to determine how reflective peripheral blood changes are relative to hepatic gene expression, we utilized the human liver-chimeric FRG mice [20]. Briefly, in this mouse strain, the selection pressure for transplanted hepatocytes is accentuated due to absence of the enzyme fumaryl acetoacetate hydrolase (FAH), which leads to an accumulation of toxic tyrosine catabolites within mouse hepatocytes. This genetically determined toxicity is preventable by oral administration of 2-(2-nitro-4-trifluoro-methylbenzoyl)-1,3-cyclohexanedione (NTBC), which blocks hydroxyphenylpyruvate dioxygenase activity upstream of FAH and therefore prevents the accumulation of hepatotoxic metabolites [21]. After withdrawal of NTBC, engrafted human hepatocytes repopulate the mouse liver; serum albumin is a reliable correlate of human hepatocyte repopulation [20], and in our mice, we saw high circulating levels of human albumin. Following reconstitution of the mouse liver with human hepatocytes we established stable HCV infection in these mice following intravenous injection of serum from a patient with HCV genotype 1a infection (Fig 4A). The mice were then treated with either vehicle control (DMSO) or a cocktail of daclatasvir, asunaprevir, and sofosbuvir. This treatment regimen induced suppression of circulating HCV within 72 hours to levels below 42 IU/mL (Fig 4B). As noted in the peripheral blood of patients receiving DAAs, there was a significant decrease in hepatic expression of CXCL10 and CXCL11, but no significant difference in RIG-I transcription (S3 Fig). We and others have shown that innate recognition of HCV in hepatocytes through dsRNA sensors (such as RIG-I) results in recruitment of mitochondrial antiviral signaling (MAVS, also known as CARDIF/IPS-1/VISA) and TRAF3 (Tumor Necrosis Factor receptor-associated factor-3) [22]. Once sufficient viral proteins have accumulated in the cytosol, HCV uses its multifunctional NS3/4A protease, essential for HCV replication, to cleave MAVS and ablate RIG-I mediated innate immune signaling [23]. MAVS is actively suppressed by HCV infection [24]. Within 14 days of triple DAA treatment, there was increased protein expression of MAVS and an increase in the protein IFITM1, a tight junction protein only induced by IFN, that inhibits HCV entry [25] (Fig 4C and S4 Fig). These findings are important because it may suggest that DAAs additionally block HCV spread among hepatocytes. A recent study of patients with chronic HCV receiving sofosbuvir-ribavirin demonstrated that SVR was associated with a decrease in hepatic expression of multiple IFN genes [18]. In contrast to that human study we found that within 14 days DAA treatment in this humanized model there are no significant changes in the expression of IFN genes, possibly due to the residual viral protein in the liver after 14 days of DAA therapy (Fig 4C) or due to the fact that these mice lack innate immune cells known to produce IFNs. Despite these differences, the transcriptional changes we document in the peripheral blood of patients receiving DAA therapy partially reflect changes that are present in the hepatic compartment of humanized mice (S3 Fig).

## Discussion

Our longitudinal analyses demonstrate that IFN-free DAA regimens in patients with chronic HCV infection result in global resetting of innate immune signaling and inflammatory pathways. Specifically, we observe a significant decrease in the transcription of the cytokine IL1β, a cytokine that is associated with innate immune activation, liver inflammation, and progression to fibrosis. Furthermore, we demonstrate a decrease in the phosphorylation of the NFκB protein which is directly associated with innate immune activation and downstream signaling. These data suggest that unlike treatment with IFNα/ribavirin, DAA therapy rapidly decreases the expression of genes associated with innate immune activation and liver damage in the peripheral blood. In support of this notion, several early studies have demonstrated that patients treated with DAA therapy have either a halt in the progression of liver fibrosis or a reversal in liver fibrosis score [26]. Our analysis of the kinetics of PBMCs transcriptional signature highlight the rapid downregulation of CXCL10, CXCL11, IFIT1, and USP18 but delayed or no significant changes in other innate immune genes such as RIGI, IFITM1, and IRF3. These differential patterns in gene expression following DAA therapy may be related to disengagement of specific HCV components and downregulation of key innate immune evasion strategies engaged by HCV to establish chronic infection [27]. It has been demonstrated that the expression level of CXCL10 in the peripheral blood during treatment with IFNα/ribavirin is associated with response to therapy [28]. In our study we find that all patients treated with DAA therapy rapidly decrease the expression of CXCL10 in the peripheral blood, suggesting that CXCL10 is not a predictor of response to DAA therapy but, rather, a marker of reestablishment of immune homeostasis following clearance of chronic HCV infection. Further, CXCL10 and CXCL11 are chemokines that are associated with recruitment of innate immune cells to the site of inflammation and liver inflammation, thus the rapid reduction in these chemokines suggest a rapid decrease in hepatic inflammation and possibly a resetting of the cellular composition in the peripheral blood. These findings corroborate our and other findings that during DAA therapy there is a rapid normalization in both the innate and adaptive immune cell phenotypes in the peripheral blood [2, 29, 30]. Because the expected outcome of current DAA treatment is SVR, we did not include patients who experienced viral relapse, which could be considered a relative limitation of the study and therefore we are unable to determine if there are any molecular characteristics that are associated with DAA non-responsiveness. However, future studies with larger cohorts focused on individuals who fail to respond to front-line DAA therapies will address these shortcomings. Transcriptional changes associated with DAA therapy versus changes that are associated with IFN-based viral cure were identified. Using this approach, we were able to highlight several key differences in the transcriptional profile in the peripheral blood between patients treated with DAAs and those treated with IFNα/ribavirin. We found that despite the rapid clearance of HCV in both cohorts we observed patients treated with IFNα/ribavirin maintained a transcriptional signature associated with innate immune activation, suggesting that DAA therapies uniquely result in the rapid return to innate immune homeostasis. One potential limitation with this analysis is that IFN treatment is well known to modulate the transcriptional levels of several genes identified in our analysis. To mitigate the direct effect of IFN we examined the transcriptional changes associated with IFN-free and IFN-containing regimens well after the end of treatment. In this analysis we found that the transcriptional differences between IFN-free and IFN-containing treatment regimens were maintained long after removal of treatment as demonstrated by persistent, elevated expression of CXCL10 and CXCL11 twenty-four weeks after removal of IFNα/ribavirin therapy. These data suggest that resolution of inflammation in the liver associated with chronic HCV infection is much more rapid in patients treated with DAA therapy than in patients treated with IFNα/ribavirin and this may be ultimately reflected in regression of liver fibrosis following HCV cure. While our transcriptional data only addressed changes in the peripheral blood of human patients, we were able to utilize a humanized mouse model to establish chronic HCV infection and also treat the infection with DAA therapy. Using this strategy, we were able to demonstrate that the transcriptional changes that occurred in the peripheral blood of patients treated with DAAs may partially, but not entirely, reflect intrahepatic transcriptional changes. Specifically, we see that the rapid reduction in the expression of CXCL10 and CXCL11 in the peripheral blood is recapitulated in the liver of humanized mice treated with a regimen of DAAs. A significant limitation with these murine studies is that residual murine hepatocytes (and non-parenchymal cells) are present in the liver, making it a human-mouse chimera. In this study we utilized mice that had >90% of the liver reconstituted with human hepatocytes, but despite this high degree of chimerism, we were unable to completely examine the transcriptional response of human hepatocytes to HCV infection or DAA therapy due to contamination with murine transcripts. Specifically, we performed RNA-seq analysis on the liver of HCV-infected mice treated with DAA therapy or DMSO control. However, contamination with murine transcripts precluded accurate analysis of the data. To counteract these problems, we selected transcripts of interest in which the primers did not detect murine transcripts. Thus, our data does not provide the complete picture of transcriptional changes in the liver following DAA therapy, but we believe our findings correlate well with the published findings from the liver of patients treated with DAAs, and the differences observed utilizing our mouse model may also be a result of the incomplete clearance of HCV protein in the liver due because of the immune-incompetent state of these humanized mice. Thus, our findings demonstrate DAA therapy rapidly reduces inflammation in the peripheral blood and liver, a finding that is not associated with IFNα/ribavirin therapy. Taken together, our study highlights not only the important transcriptional pathways associated with HCV cure but also the molecular pathways that are associated with resolution of hepatic inflammation.

## Supporting information

S1 Table Patient demographics. **A**. DAA cohort 1. Top 9 patient PBMC were used for microarray analysis and RT-PCR while the bottom 15 patients were used for RT-PCR of PBMC or Paxgene tubes. **B**. DAA cohort 2. Patient PBMCs were used for RT-PCR.
(DOCX)

S1 Fig Confirmation of microarray transcriptional changes. RT-PCR of selected genes with significant changes at the end of treatment (EOT) compared to pretreatment. N = 9. *P<0.05.
(DOCX)

S2 Fig Comparison of transcriptional changes between DAA and IFNα/Ribavirin therapy. Paired t-test on both DAA and the GSE11342 IFN dataset using the 70 day timepoint on the former using those individuals in the IFN dataset that completely cleared the virus by day 70. All uncharacterized genes (without Ref Seq #) were eliminated from both gene lists, leaving 38,598 out of 53,617 genes for DAA and 21,174 out of 22,284 for GSE11342 (IFN dataset). **A**. X-axis is the mean difference (mean log2 expression Post—mean log2 expression Pre) of the DAA treatment, Y axis is the mean difference (mean log2 expression Post—mean log2 expression Pre) of the IFN dataset. The color code is the mean difference of the DAA samples minus the mean difference of the interferon samples (blue-grey would be more decreased in DAA compared to interferon, red is the opposite). The top 10 genes with the largest and smallest positive and negative values respectively for the mean difference of DAA- Interferon (this is the color code values) were labeled. **B**. Taking the 520 genes that were different between Post vs Pre- treatment with DAA at a P<0.01 (paired t-test) and merged any available data from the interferon Post vs Pre- samples for all common genes. This left 214 genes different in pre vs. post DAA treatment that were also measured in the interferon dataset.
(DOCX)

S3 Fig Transcriptional changes in the liver of humanized mice treated with DAAs. RT-PCR expression of CXCL10, CXCL11 and RIG-I in mice chronically infected with HCV genotype 1 treated with DMSO (Black Bars) or DAAs (White Bars) for 14 days. *P<0.05.
(DOCX)

S4 Fig Restoration of innate immune signaling in the livers of mice treated with DAAs. Representative images of MAVS (red), NS3 (green), DAPI (blue) **A** and merged or IRF3 (red), IFITM1 (green), DAPI (blue) and merged **B**. Scale = 50μm.
(DOCX)

## References

[1] Everson G, Cooper C, Hezode C, Shiffman ML, Yoshida E, Beltran-Jaramillo T, et al DAUPHINE: a randomized phase II study of danoprevir/ritonavir plus peginterferon alpha-2a/ribavirin in HCV genotypes 1 or 4. Liver international: official journal of the International Association for the Study of the Liver. 2015;35(1):108–19. doi: 10.1111/liv.12471 . 24517252 10.1111/liv.12471

[2] Burchill MA, Golden-Mason L, Wind-Rotolo M, Rosen HR. Memory re-differentiation and reduced lymphocyte activation in chronic HCV-infected patients receiving direct-acting antivirals. Journal of viral hepatitis. 2015;22(12):983–91. doi: 10.1111/jvh.12465 . 26482547 10.1111/jvh.12465

[3] Conjeevaram HS, Fried MW, Jeffers LJ, Terrault NA, Wiley-Lucas TE, Afdhal N, et al Peginterferon and ribavirin treatment in African American and Caucasian American patients with hepatitis C genotype 1. Gastroenterology. 2006;131(2):470–7. doi: 10.1053/j.gastro.2006.06.008 . 16890601 10.1053/j.gastro.2006.06.008

[4] Burton JR Jr., Klarquist J, Im K, Smyk-Pearson S, Golden-Mason L, Castelblanco N, et al Prospective analysis of effector and regulatory CD4+ T cells in chronic HCV patients undergoing combination antiviral therapy. J Hepatol. 2008;49(3):329–38. doi: 10.1016/j.jhep.2008.05.020 . 18644644 10.1016/j.jhep.2008.05.020

[5] McMahan RH, Porsche CE, Edwards MG, Rosen HR. Free Fatty Acids Differentially Downregulate Chemokines in Liver Sinusoidal Endothelial Cells: Insights into Non-Alcoholic Fatty Liver Disease. PLoS One. 2016;11(7):e0159217 doi: 10.1371/journal.pone.0159217 . 27454769 10.1371/journal.pone.0159217 PMC4959750

[6] Ellis EC, Naugler WE, Parini P, Mork LM, Jorns C, Zemack H, et al Mice with chimeric livers are an improved model for human lipoprotein metabolism. PLoS One. 2013;8(11):e78550 doi: 10.1371/journal.pone.0078550 . 24223822 10.1371/journal.pone.0078550 PMC3817217

[7] Azuma H, Paulk N, Ranade A, Dorrell C, Al-Dhalimy M, Ellis E, et al Robust expansion of human hepatocytes in Fah-/-/Rag2-/-/Il2rg-/- mice. Nat Biotechnol. 2007;25(8):903–10. doi: 10.1038/nbt1326 . 17664939 10.1038/nbt1326 PMC3404624

[8] Horner SM, Gale M Jr. Regulation of hepatic innate immunity by hepatitis C virus. Nat Med. 2013;19(7):879–88. doi: 10.1038/nm.3253 . 23836238 10.1038/nm.3253 PMC4251871

[9] Tsuchida T, Friedman SL. Mechanisms of hepatic stellate cell activation. Nat Rev Gastroenterol Hepatol. 2017 doi: 10.1038/nrgastro.2017.38 . 28487545 10.1038/nrgastro.2017.38

[10] Rosen HR. Emerging concepts in immunity to hepatitis C virus infection. J Clin Invest. 2013;123(10):4121–30. doi: 10.1172/JCI67714 . 24084744 10.1172/JCI67714 PMC3784533

[11] Negash AA, Ramos HJ, Crochet N, Lau DT, Doehle B, Papic N, et al IL-1beta production through the NLRP3 inflammasome by hepatic macrophages links hepatitis C virus infection with liver inflammation and disease. PLoS Pathog. 2013;9(4):e1003330 doi: 10.1371/journal.ppat.1003330 . 23633957 10.1371/journal.ppat.1003330 PMC3635973

[12] Miura K, Kodama Y, Inokuchi S, Schnabl B, Aoyama T, Ohnishi H, et al Toll-like receptor 9 promotes steatohepatitis by induction of interleukin-1beta in mice. Gastroenterology. 2010;139(1):323–34 e7. doi: 10.1053/j.gastro.2010.03.052 . 20347818 10.1053/j.gastro.2010.03.052 PMC4631262

[13] Gieling RG, Wallace K, Han YP. Interleukin-1 participates in the progression from liver injury to fibrosis. Am J Physiol Gastrointest Liver Physiol. 2009;296(6):G1324–31. doi: 10.1152/ajpgi.90564.2008 . 19342509 10.1152/ajpgi.90564.2008 PMC2697947

[14] Dienstag JL, McHutchison JG. American Gastroenterological Association technical review on the management of hepatitis C. Gastroenterology. 2006;130(1):231–64; quiz 14–7. doi: 10.1053/j.gastro.2005.11.010 . 16401486 10.1053/j.gastro.2005.11.010

[15] Schaefer M, Capuron L, Friebe A, Diez-Quevedo C, Robaeys G, Neri S, et al Hepatitis C infection, antiviral treatment and mental health: a European expert consensus statement. J Hepatol. 2012;57(6):1379–90. doi: 10.1016/j.jhep.2012.07.037 . 22878466 10.1016/j.jhep.2012.07.037

[16] Schaefer M, Sarkar R, Knop V, Effenberger S, Friebe A, Heinze L, et al Escitalopram for the prevention of peginterferon-alpha2a-associated depression in hepatitis C virus-infected patients without previous psychiatric disease: a randomized trial. Ann Intern Med. 2012;157(2):94–103. doi: 10.7326/0003-4819-157-2-201207170-00006 . 22801672 10.7326/0003-4819-157-2-201207170-00006

[17] Fried MW. Side effects of therapy of hepatitis C and their management. Hepatology. 2002;36(5 Suppl 1):S237–44. doi: 10.1053/jhep.2002.36810 . 12407599 10.1053/jhep.2002.36810

[18] Meissner EG, Wu D, Osinusi A, Bon D, Virtaneva K, Sturdevant D, et al Endogenous intrahepatic IFNs and association with IFN-free HCV treatment outcome. J Clin Invest. 2014;124(8):3352–63. doi: 10.1172/JCI75938 . 24983321 10.1172/JCI75938 PMC4109554

[19] Taylor MW, Tsukahara T, McClintick JN, Edenberg HJ, Kwo P. Cyclic changes in gene expression induced by Peg-interferon alfa-2b plus ribavirin in peripheral blood monocytes (PBMC) of hepatitis C patients during the first 10 weeks of treatment. J Transl Med. 2008;6:66 doi: 10.1186/1479-5876-6-66 . 18986530 10.1186/1479-5876-6-66 PMC2613871

[20] Bissig KD, Wieland SF, Tran P, Isogawa M, Le TT, Chisari FV, et al Human liver chimeric mice provide a model for hepatitis B and C virus infection and treatment. J Clin Invest. 2010;120(3):924–30. Epub 2010/02/25. doi: 10.1172/JCI40094 . 20179355 10.1172/JCI40094 PMC2827952

[21] Grompe M, al-Dhalimy M, Finegold M, Ou CN, Burlingame T, Kennaway NG, et al Loss of fumarylacetoacetate hydrolase is responsible for the neonatal hepatic dysfunction phenotype of lethal albino mice. Genes Dev. 1993;7(12A):2298–307. . 8253378 10.1101/gad.7.12a.2298

[22] Arnaud N, Dabo S, Akazawa D, Fukasawa M, Shinkai-Ouchi F, Hugon J, et al Hepatitis C virus reveals a novel early control in acute immune response. PLoS Pathog. 2011;7(10):e1002289 doi: 10.1371/journal.ppat.1002289 . 22022264 10.1371/journal.ppat.1002289 PMC3192838

[23] Horner SM, Liu HM, Park HS, Briley J, Gale M Jr. Mitochondrial-associated endoplasmic reticulum membranes (MAM) form innate immune synapses and are targeted by hepatitis C virus. Proc Natl Acad Sci U S A. 2011;108(35):14590–5. doi: 10.1073/pnas.1110133108 . 21844353 10.1073/pnas.1110133108 PMC3167523

[24] Li XD, Sun L, Seth RB, Pineda G, Chen ZJ. Hepatitis C virus protease NS3/4A cleaves mitochondrial antiviral signaling protein off the mitochondria to evade innate immunity. Proc Natl Acad Sci U S A. 2005;102(49):17717–22. doi: 10.1073/pnas.0508531102 . 16301520 10.1073/pnas.0508531102 PMC1308909

[25] Wilkins C, Woodward J, Lau DT, Barnes A, Joyce M, McFarlane N, et al IFITM1 is a tight junction protein that inhibits hepatitis C virus entry. Hepatology. 2013;57(2):461–9. doi: 10.1002/hep.26066 . 22996292 10.1002/hep.26066 PMC3566288

[26] Bachofner JA, Valli PV, Kroger A, Bergamin I, Kunzler P, Baserga A, et al Direct antiviral agent treatment of chronic hepatitis C results in rapid regression of transient elastography and fibrosis markers fibrosis-4 score and aspartate aminotransferase-platelet ratio index. Liver international: official journal of the International Association for the Study of the Liver. 2017;37(3):369–76. doi: 10.1111/liv.13256 . 27678216 10.1111/liv.13256

[27] Horner SM. Activation and evasion of antiviral innate immunity by hepatitis C virus. J Mol Biol. 2014;426(6):1198–209. doi: 10.1016/j.jmb.2013.10.032 . 24184198 10.1016/j.jmb.2013.10.032 PMC4431647

[28] Kurelac I, Lepej SZ, Grlgic I, Gorenec L, Papic N, Dusek D, et al Chemokine CXCL10 at week 4 of treatment predicts sustained virological response in patients with chronic hepatitis C. J Interferon Cytokine Res. 2012;32(8):386–91. doi: 10.1089/jir.2012.0006 . 22799464 10.1089/jir.2012.0006 PMC3422056

[29] Spaan M, van Oord G, Kreefft K, Hou J, Hansen BE, Janssen HL, et al Immunological Analysis During Interferon-Free Therapy for Chronic Hepatitis C Virus Infection Reveals Modulation of the Natural Killer Cell Compartment. The Journal of infectious diseases. 2016;213(2):216–23. doi: 10.1093/infdis/jiv391 . 26223768 10.1093/infdis/jiv391

[30] Serti E, Chepa-Lotrea X, Kim YJ, Keane M, Fryzek N, Liang TJ, et al Successful Interferon-Free Therapy of Chronic Hepatitis C Virus Infection Normalizes Natural Killer Cell Function. Gastroenterology. 2015;149(1):190–200 e2. doi: 10.1053/j.gastro.2015.03.004 . 25754160 10.1053/j.gastro.2015.03.004 PMC4523392

