# Supplementary material for: Rapid reversal of innate immune dysregulation in blood of patients and livers of humanized mice with HCV following DAA therapy
Source: PLoS One. 2017 Oct 17;12(10):e0186213. doi: 10.1371/journal.pone.0186213 (PMC5645093; doi:10.1371/journal.pone.0186213)
Supplement: S1 Table — A. DAA cohort 1. Top 9 patient PBMC were used for microarray analysis and RT-PCR while the bottom 15 patients were used for RT-PCR of PBMC or Paxgene tubes. B. DAA cohort 2. Patient PBMCs were used for RT-PCR. (DOCX) [file pone.0186213.s001.docx]

**A.**

**B.**

**Supplemental Table 1.** Patient demographics

**A.** DAA cohort 1. Top 9 patient PBMC were used for microarray analysis and RT-PCR while the bottom 15 patients were used for RT-PCR of PBMC or Paxgene tubes. **B.**  DAA cohort 2. Patient PBMCs were used for RT-PCR
