# Supplementary figures and images for: Rapid reversal of innate immune dysregulation in blood of patients and livers of humanized mice with HCV following DAA therapy
Source: PLoS One. 2017 Oct 17;12(10):e0186213. doi: 10.1371/journal.pone.0186213 (PMC5645093; doi:10.1371/journal.pone.0186213)

**A.**

**
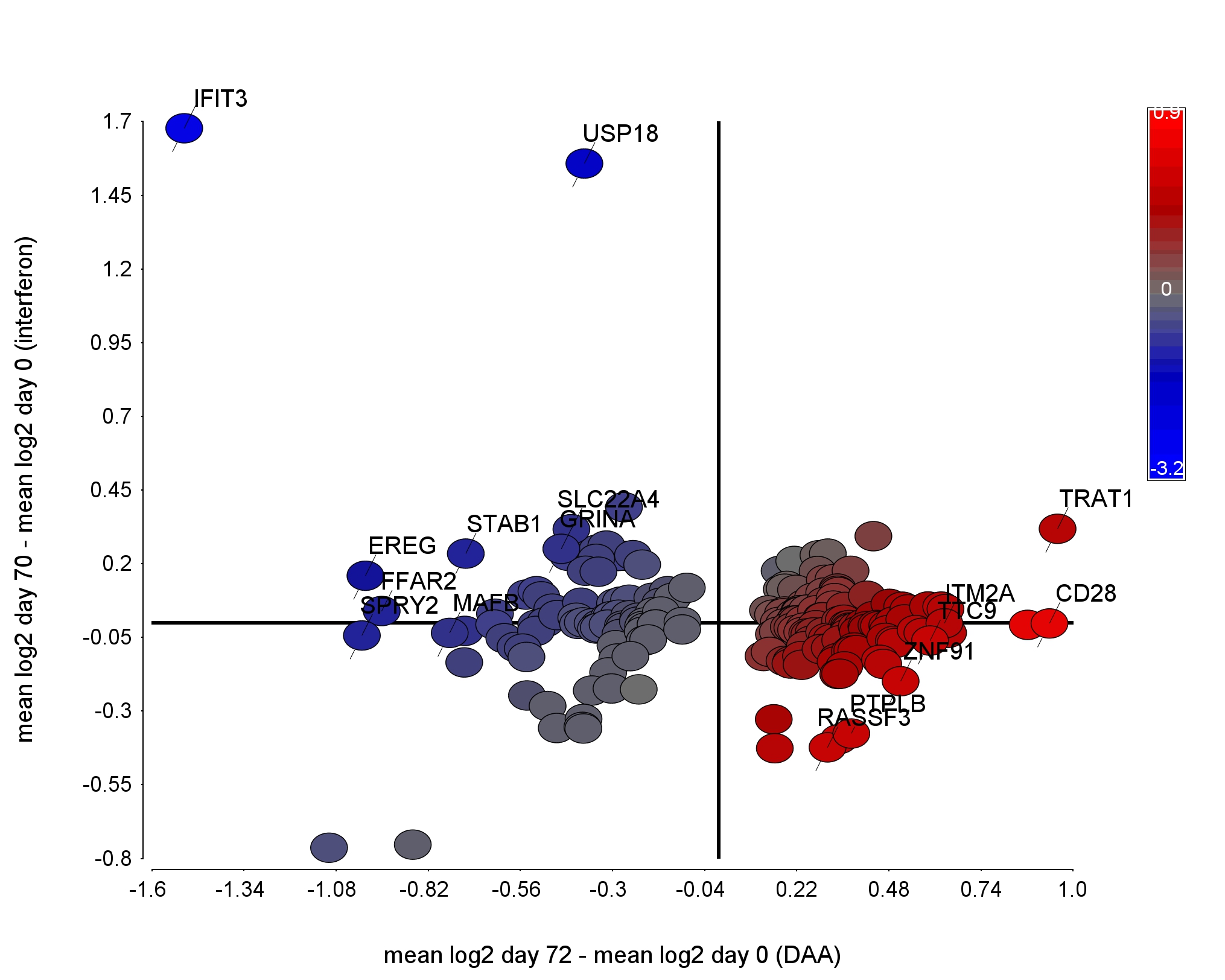
**

**B.**

**
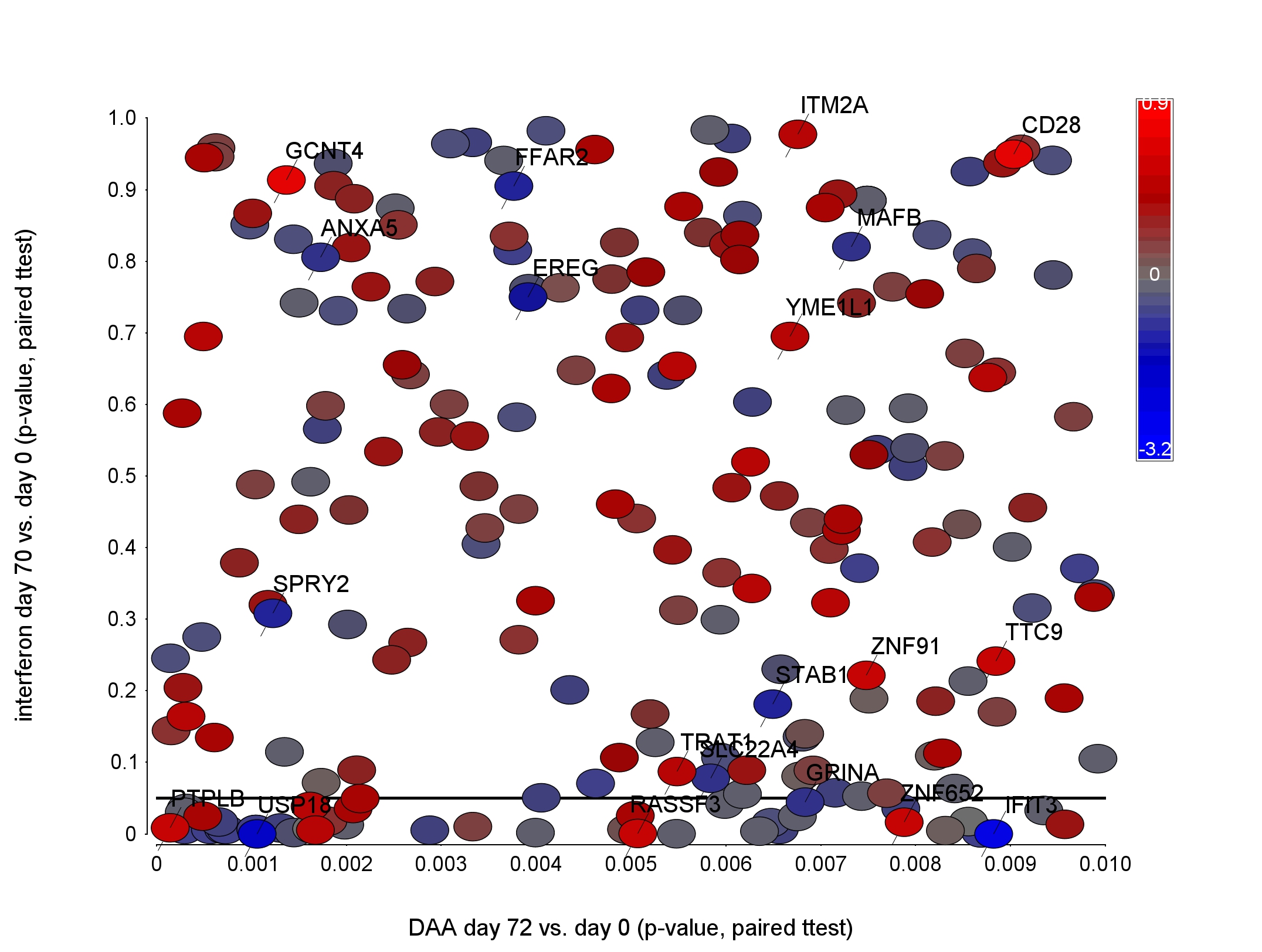
**

Supplement: S2 Fig — Paired t-test on both DAA and the GSE11342 IFN dataset using the 70 day timepoint on the former using those individuals in the IFN dataset that completely cleared the virus by day 70. All uncharacterized genes (without Ref Seq #) were eliminated from both gene lists, leaving 38,598 out of 53,617 genes for DAA and 21,174 out of 22,284 for GSE11342 (IFN dataset). A. X-axis is the mean difference (mean log2 expression Post—mean log2 expression Pre) of the DAA treatment, Y axis is the mean difference (mean log2 expression Post—mean log2 expression Pre) of the IFN dataset. The color code is the mean difference of the DAA samples minus the mean difference of the interferon samples (blue-grey would be more decreased in DAA compared to interferon, red is the opposite). The top 10 genes with the largest and smallest positive and negative values respectively for the mean difference of DAA- Interferon (this is the color code values) were labeled. B. Taking the 520 genes that were different between Post vs Pre- treatment with DAA at a P<0.01 (paired t-test) and merged any available data from the interferon Post vs Pre- samples for all common genes. This left 214 genes different in pre vs. post DAA treatment that were also measured in the interferon dataset. (DOCX) [file pone.0186213.s003.docx]

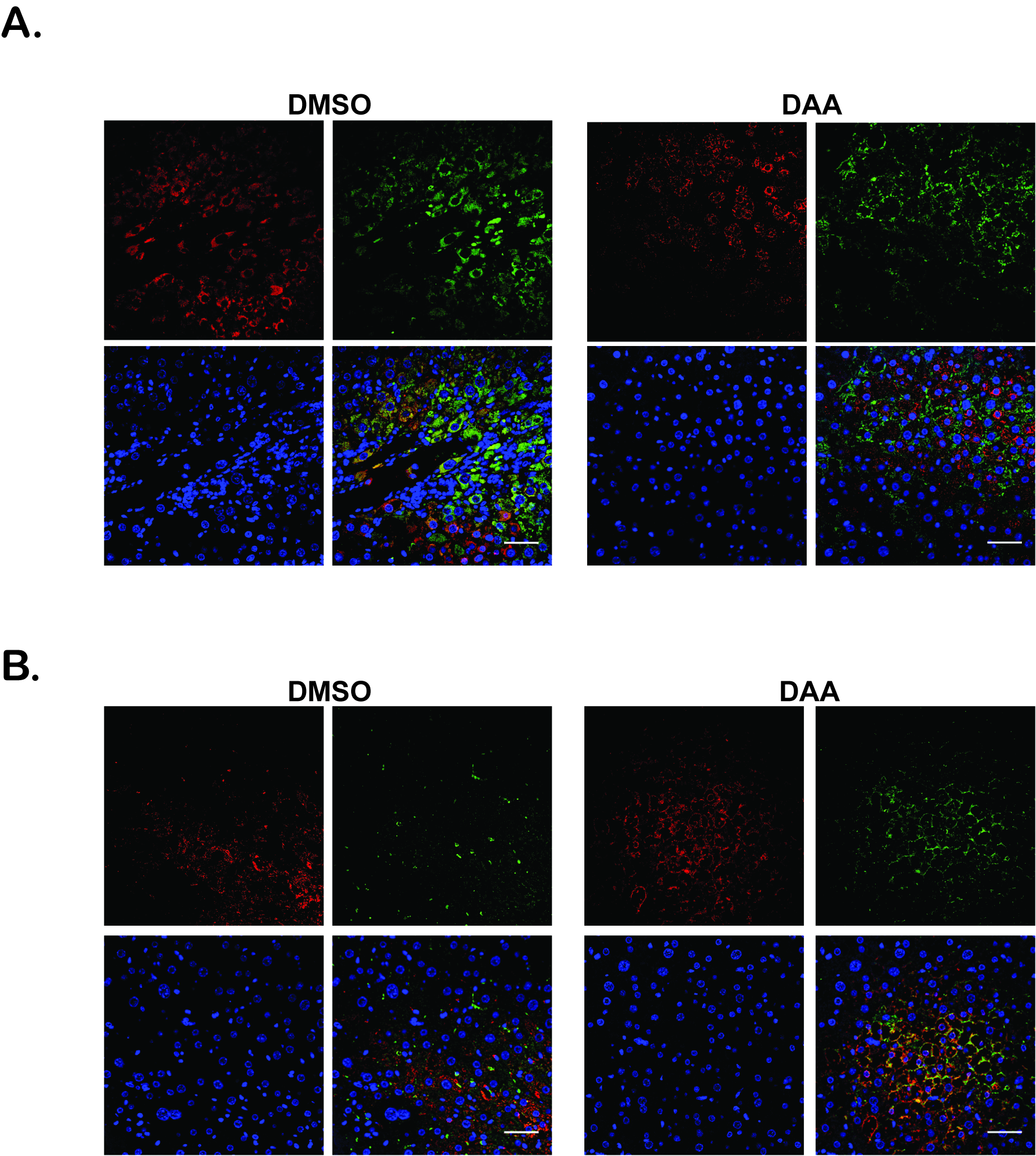

Supplement: S4 Fig — Representative images of MAVS (red), NS3 (green), DAPI (blue) A and merged or IRF3 (red), IFITM1 (green), DAPI (blue) and merged B. Scale = 50μm. (DOCX) [file pone.0186213.s005.docx]
