# Supplementary material for: Rapid reversal of innate immune dysregulation in blood of patients and livers of humanized mice with HCV following DAA therapy
Source: PLoS One. 2017 Oct 17;12(10):e0186213. doi: 10.1371/journal.pone.0186213 (PMC5645093; doi:10.1371/journal.pone.0186213)
Supplement: Supplementary file 3 [file pone.0186213.s002.docx]

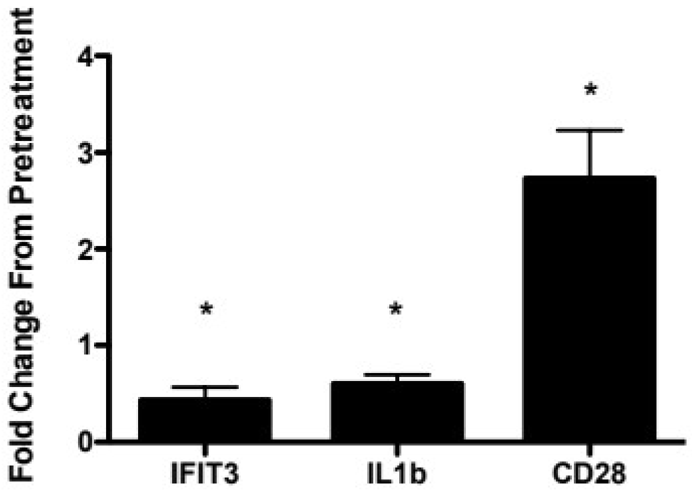
Fold Change From Pretreatment
4
3
2
1
0
*
*
*
IFIT3
IL1b
CD28
