# Supplementary material for: Rapid reversal of innate immune dysregulation in blood of patients and livers of humanized mice with HCV following DAA therapy
Source: PLoS One. 2017 Oct 17;12(10):e0186213. doi: 10.1371/journal.pone.0186213 (PMC5645093; doi:10.1371/journal.pone.0186213)
Supplement: Supplementary file 4 [file pone.0186213.s004.docx]

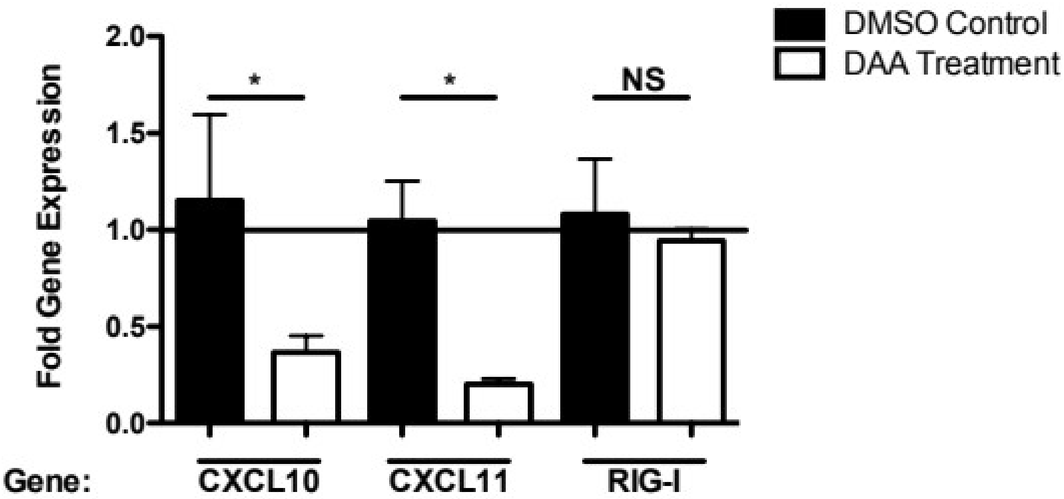
DMSO Control
DAA Treatment
Fold Gene Expression
2.0
1.5
1.0
0.5
0.0
*
*
NS
Gene:
CXCL10
CXCL11
RIG-I
